# Public Officials’ Engagement on Social Media During the Rollout of the COVID-19 Vaccine: Content Analysis of Tweets

**DOI:** 10.2196/41582

**Published:** 2023-07-20

**Authors:** Husayn Marani, Melodie Yunju Song, Margaret Jamieson, Monika Roerig, Sara Allin

**Affiliations:** 1 Institute of Health Policy, Management and Evaluation Dalla Lana School of Public Health University of Toronto Toronto, ON Canada; 2 North American Observatory on Health Systems and Policies University of Toronto Toronto, ON Canada; 3 Centre for Vaccine Preventable Diseases Dalla Lana School of Public Health University of Toronto Toronto, ON Canada

**Keywords:** Twitter, COVID-19, vaccines, sentiment analysis, public officials

## Abstract

**Background:**

Social media is an important way for governments to communicate with the public. This is particularly true in times of crisis, such as the COVID-19 pandemic, during which government officials played a strong role in promoting public health measures such as vaccines.

**Objective:**

In Canada, provincial COVID-19 vaccine rollout was delivered in 3 phases aligned with federal government COVID-19 vaccine guidance for priority populations. In this study, we examined how Canadian public officials used Twitter to engage with the public about vaccine rollout and how this engagement has shaped public response to vaccines across jurisdictions.

**Methods:**

We conducted a content analysis of tweets posted between December 28, 2020, and August 31, 2021. Leveraging the social media artificial intelligence tool Brandwatch Analytics, we constructed a list of public officials in 3 jurisdictions (Ontario, Alberta, and British Columbia) organized across 6 public official types and then conducted an English and French keyword search for tweets about vaccine rollout and delivery that mentioned, retweeted, or replied to the public officials. We identified the top 30 tweets with the highest impressions in each jurisdiction in each of the 3 phases (approximately a 26-day window) of the vaccine rollout. The metrics of engagement (impressions, retweets, likes, and replies) from the top 30 tweets per phase in each jurisdiction were extracted for additional annotation. We specifically annotated sentiment toward public officials’ vaccine responses (ie, positive, negative, and neutral) in each tweet and annotated the type of social media engagement. A thematic analysis of tweets was then conducted to add nuance to extracted data characterizing sentiment and interaction type.

**Results:**

Among the 6 categories of public officials, 142 prominent accounts were included from Ontario, Alberta, and British Columbia. In total, 270 tweets were included in the content analysis and 212 tweets were direct tweets by public officials. Public officials mostly used Twitter for information provision (139/212, 65.6%), followed by horizontal engagement (37/212, 17.5%), citizen engagement (24/212, 11.3%), and public service announcements (12/212, 5.7%). Information provision by government bodies (eg, provincial government and public health authorities) or municipal leaders is more prominent than tweets by other public official groups. Neutral sentiment accounted for 51.5% (139/270) of all the tweets, whereas positive sentiment was the second most common sentiment (117/270, 43.3%). In Ontario, 60% (54/90) of the tweets were positive. Negative sentiment (eg, public officials criticizing vaccine rollout) accounted for 12% (11/90) of all the tweets.

**Conclusions:**

As governments continue to promote the uptake of the COVID-19 booster doses, findings from this study are useful in informing how governments can best use social media to engage with the public to achieve democratic goals.

## Introduction

### Background

With the global usership of popular social media platforms such as Twitter, Facebook, and Instagram in the billions, it is no longer a choice but a necessity for government institutions and public officials therein to have a social media presence [1]. For better or worse, the proliferation of social media in recent decades has transformed how governments achieve various public policy goals [2]. This has given rise to e-governance, whereby communication technologies such as social media are leveraged by government institutions to improve information exchange (improving transparency), enhance citizen engagement in democratic processes (increasing participation), and foster collaboration between government institutions and constituents to improve government-related activities (strengthening collaboration) [3].

Numerous frameworks exist to typify the ways in which government institutions interact with the public over social media to achieve various democratic goals. The seminal framework by Mergel [4] organizes the abovementioned goals into a 3-category framework called the *open government framework of interpreting the impact of social media interactions* (herein *government interaction framework*); the framework describes public sector’s tactical use of social media to push information, pull information, and network. Each of these tactics relate to improving transparency, increasing participation, and strengthening collaboration, respectively [4]. Additional goals, such as public service delivery [3], self-presentation and marketing [5], and facilitating local social transactions [6], have yielded adaptations to the framework by Mergel [4]. Broadly, these frameworks are useful to answer important questions about how government institutions may interact with the public over social media in specific contexts, for example, by responding, retweeting—which could imply engagement on Twitter, or mentioning others [7]. These frameworks are also useful for evaluating the effectiveness of public response to government social media communication. For example, indicators such as user sentiment [8], audience engagement (volume of *likes*, *comments*, and *shares* by the public) [9], and emotion in textual responses [10] indicate public receptivity toward policy rollout and government responses [11].

Recently, we have seen government institutions leveraging social media to engage the public during times of crisis, such as weather catastrophes and viral epidemics over short periods [12-14]. Studies on these contexts are largely oriented toward local levels of e-governance, as local governments are the closest to citizens and can more effectively achieve interaction goals, such as participation and public service delivery [3,15]. However, there is a relative dearth of the literature concerning how social media is used by government institutions at the national, regional, and local levels during times of crisis. Filling this gap is important in settings where crucial decision-making concerning crises is derived across multiple levels of government—such as in the case of Canada.

### Objectives

The COVID-19 pandemic has seen a significant use of social media by the government to achieve various goals, including mitigating the transmission of COVID-19 through information dissemination, encouraging behavioral changes, and promoting the availability of community-based supports and resources [16,17]. Given the decentralized structure of the Canadian health care system, national (federal) and subnational (provincial and territorial) governments played both independent and collaborative roles in responding to the COVID-19 pandemic. Indeed, recent research from Canada has shown that approaches to health communication over social media regarding the risks of COVID-19 differ between jurisdictions and depend in part on the local burden of COVID-19 [18]. This suggests an important responsibility of regional and local governments (provinces, territories, and municipalities) in delivering messaging tailored to the levels of risk audiences are encountering in each geographic context.

Of particular interest to our study are the activities and engagement of decision makers and policy makers, specifically elected public officials, in achieving the goal of population uptake of the COVID-19 vaccine. The vaccine rollout has generated significant policy and public debate in Canada. Achieving high vaccination coverage was seen as the pathway to normalcy, starting with the end of COVID-19 *lockdowns* across the country [19]. Although the federal government is responsible for vaccine procurement and distribution to the provinces and territories, provincial and territorial governments have been responsible for vaccine rollout. In December 2020, all 10 provinces and 3 territories took a phased approach to first-dose vaccine rollout that generally aligned with the advice from the National Advisory Committee on Immunization [20]. Rollout started with health care workers (HCWs), persons living in high-risk congregate settings such as residential long-term care homes, older adults (aged >75 years), and Indigenous communities. This was generally followed by a phased plan based on age cohorts and the presence of health-related risk factors [21,22]. The second dose rollout followed a similar structure, but a more concerted effort was made to prioritize COVID-19 *hotspots* calculated by case positivity rates in postal districts; for instance, the Ministry of Health in Ontario devised an age-based vaccine rollout strategy that prioritized those residing in communal *hotspots* [23,24].

Emerging scholarship from the United States shows that leaders across the political spectrum have used social media, in particular Twitter, to promote uptake of the COVID-19 vaccine [25]. However, little is known about how Canadian public officials engaged with the public through social media during the COVID-19 pandemic. With 77.6% of the Canadians above the age of 15 years regularly using social media and 25.2% of the Canadians on at least 3 social media platforms in 2018 [26], exposure to vaccine-related misinformation and disinformation has threatened public confidence in the vaccine and overall uptake [27]. Accordingly, Canadian public officials have played an important role not only in combating a historical propensity among Canadian populations to refuse vaccines [28], which was documented near the start of the COVID-19 vaccine rollout [29], but also in combating antivaccine misinformation and disinformation on social media, which has been shown to be predictive of vaccine hesitancy [30]. The purpose of this exploratory study was to gain insight into how public officials across federal and provincial governments leveraged social media to communicate with the public about the COVID-19 vaccine rollout, particularly across the 3 phases of rollout when clear public communication was especially important.

## Methods

### Overview

To identify social media posts, we used Brandwatch Analytics (henceforth Brandwatch), a social media intelligence tool that uses proprietary artificial intelligence to extract and analyze social media data from various social media platforms. Previously used to conduct textual analyses of Twitter data in the context of the COVID-19 pandemic [31], its enterprise Twitter application programming interface has 2 advantages. First, it makes it possible to retrieve retrospective data. Second, it retrieves a higher percentage of tweets within a given time interval, presenting a more representative set of tweets over time. We focused on Twitter for consistency with previous scholarship on health messaging and crisis communication by public officials [18,32] and because Twitter is the most used social media platform by public officials and departments across the Government of Canada to communicate and engage with the public [33].

### Data Collection

To obtain tweets about vaccine rollout, we developed an English and French keyword query using a combination of truncation and Boolean operators and wildcards (* and ?), which were used to identify posts containing common root words and letter substitutions, respectively. This query was used to capture conversations about vaccine rollout in Canada between December 28, 2020, and August 31, 2021 (Textbox 1). This query was run twice: first, to canvas Twitter participation of public officials in Canada and second, to derive a sample of tweets for content analysis.

**Keyword query to capture tweets about vaccine rollout.**

(((vaccin* OR vax* OR immuniz* OR immunis*) AND (distribu* OR allocat* OR roll-out OR “roll out” OR deliver* OR provid* OR provision* OR administer* OR administr* OR livraison OR apporter OR alloue*)))

### Canvassing Twitter Participation of Public Officials in Canada

A first run of the query showed that most of the conversation driving vaccine rollout across Canada (106,834/124,081, 86.1%) occurred in 3 provinces: Ontario (80,156/124,081, 64.6% of the total tweets), Alberta (15,137/124,081, 12.2%), and British Columbia (BC; 11,539/124,081, 9.3%). Unsurprisingly, these are the most Twitter-engaged provinces [34] and are among the most populous provinces in Canada. To narrow down tweets about vaccine rollout posted by, or mentioning, public officials across Canada, we used results from this query to identify the top 20 public officials, irrespective of being *verified* by Twitter, with the highest cumulative engagement (which we estimated based on total impressions) in each of the 3 provinces. This list aimed to supplement an a priori list of public officials generated by the study team of any public officials, including the organizations or provincial government and public health authorities to which they belong, across Canada, who are on Twitter and who have been involved in broad decision-making related to COVID-19 vaccine procurement and rollout. Public officials were organized across 6 categories of public official types (Textbox 2) inspired by another study with a similar organizational framework [18]. In total, 142 user accounts of public officials were included in our second run of the query (Multimedia Appendix 1).

**Six categories of public officials involved in vaccine rollout decision-making.**

1. *First ministers (premiers; n=15)*: this includes publicly elected federal and provincial and territorial heads of government, including the Prime Minister, Deputy Prime Minister, and provincial and territorial Premiers.
2. *Ministers of Health (n=16)*: this includes the official, acting, interim, and deputy Ministers of Health in every province and territory.
3. *Chief Medical Officers of Health (n=6)*: this includes verified Twitter accounts of the Chief Public Health Officer of Canada and provincial Chief Public Health and Medical Officers of Health.
4. *Government bodies (n=53)*: this includes official organizational accounts of the Federal Government (ie, Government of Canada); federal public health authorities (ie, Health Canada and Public Health Agency of Canada); provincial governments (ie, Government of New Brunswick) and provincial public health authorities (ie, Saskatchewan Health Authority) who are involved in vaccine rollout and decision-making.
5. *Municipal officials (n=14)*: this includes publicly elected officials at the municipal level, including mayors of capital cities (eg, Toronto, Ottawa, Vancouver, and Calgary).
6. *Other key public officials (n=38)*: this includes elected members of parliament and PT legislative assemblies, which captures Ministers with any form of engagement in vaccine rollout (eg, Minister of Public Services and Procurement) who are not Ministers of Health.

### Identifying Public Official Participation on Social Media During the Initiation of Vaccine Rollout Phase Changes in Alberta, BC, and Ontario

We ran our query (Textbox 1) again, this time in combination with the usernames of all 142 public officials identified (Multimedia Appendix 2). This search yielded 133,155 tweets geotagged by Brandwatch as having originated in Canada that mentioned, retweeted, and replied to our list of public officials. Similar to our first query, most tweets (106,834/133,155, 80.23%) came from 3 provinces: Ontario (80,201/106,834, 75.07%), Alberta (15,096/106,834, 14.13%), and BC (11,537/106,834, 10.8%). We selected these 3 provinces for content analysis because these are the 3 most populous provinces in Canada, bar Quebec, which has a low Twitter participation from public officials based on our canvassing activity above (see the *Canvassing Twitter Participation of Public Officials in Canada* section)

To identify the tweets that were driving the conversation during each phase of the vaccine rollout, we sorted all tweets in each province by phase (Multimedia Appendix 3 [35]), and then sorted tweets by impressions (highest to lowest). To ensure that the tweets were temporally consistent with the phase changes, we only extracted tweets that were tweeted 5 days from the onset of the phase change announcement up to 3 weeks following the announcement, for a total of 26 days. For each 26-day period, we extracted the top 30 tweets per phase in each province, resulting in 90 tweets per province and 270 tweets in total. A complete data collection flowchart is presented in Multimedia Appendix 4. The 270 tweets represent original tweets by public officials, quoted tweets (eg, a retweet embedded with personal commentary above the @publicofficial’s original tweet), retweets (*RT@publicofficial*), and tweets that mention or reply to public officials (*@publicofficial*). As filtering ad-based tweets was not a function made available to us in Brandwatch, and sponsored content does not require public disclosure, the collected tweets driving conversations about vaccine rollout may include ad-based or sponsored tweets.

### Data Extraction and Analysis

We conducted a content analysis of 270 Canadian-geotagged tweets with the highest impressions posted by or engaging with (ie, tagging, mentioning and replying to, or quoting) the 142 public officials accounts. Tweets were divided evenly among the 4 authors (HM, MYS, MJ, and MR) for extraction, sentiment analysis, and content analysis. In summary, the extracted criteria included the text of each tweet, any URLs in the tweet, metrics of engagement (impressions, retweets, likes, replies, and quoted retweets), province from which the tweet was derived, date of tweet, and interaction type. We also manually conducted sentiment analysis of the 270 tweets to determine whether a tweet expressed a positive, negative, or neutral stance toward the process and delivery of the vaccine rollout. A total of 4 coders (HM, MYS, MJ, and MR) participated in the coding; 3 coders were assigned to code the same tweet for all 270 tweets for both sentiment analysis and content analysis, and any discrepancies were resolved by discussion by all authors (HM, MYS, MJ, MR, and SA) if the 3 coders could not reach an agreement.

The seminal framework that Mergel [4] proposed organizes public sector social media interaction types (*push*, *pull*, and *network*) based on observations of the US federal government interacting with the public in response to the Obamacare website crisis. The *public service social media interaction framework* by Criado and Villodre [3] modified the framework by Mergel [4] after testing the framework on tweets collected in localized city councils in 4 European countries. To represent the bulk of public sector interaction, this framework used the term *public service delivery* corresponding to *networking*, *information provision* corresponding to *push*, and *citizen* *interaction* corresponding to *pull* by Mergel [4].

Our content analysis of public officials’ tweets regarding the COVID-19 vaccine generated an adapted *push and pull* framework that builds on Mergel [4] and Criado and Villodre [3]. In our framework, push-1 (*information provision*) and push-2 (*public* *service* *announcement*) interaction types complement the framework by Criado and Villodre [3] to differentiate the provision of critical updates (push-1: information provision) versus announcements about public service availability, including vaccine eligibility (push-2: public service announcement). Pull-1 (*citizen engagement*) and pull-2 (*public official* *engagement*) interaction types reflect nuances across provinces and the need to elevate communication between public officials with each other over society into its own category. Taken together, the modified framework captures all 4 public sector interaction types in large Canadian provinces.

We then assigned a code (ie, *push 1*, *push 2*, *pull 1*, and *pull 2*) to each tweet based on the public service social media interaction framework [3] inspired by Mergel [4] (Textbox 3) that organizes how public officials interact with members of the public over social media to achieve public policy objectives. To understand how well tweets by each category of public officials were endorsed, we calculated an endorsement ratio derived from the number of likes received divided by the number of impressions (views) for each tweet. The endorsement ratio is a value between 0 and 1, with a higher ratio indicating a higher content-specific endorsement on Twitter.

**Types of public officials’ social media interaction that may increase vaccine uptake (modified based on the public service social media interaction frameworks of Criado and Villodre and Mergel).**

**Push 1: information provision**

- Refers to one-way social media posting activities that disseminate data, and have broad aims to increase transparency, accountability, and citizen trust.

**Push 2: public service announcement**

- Refers to one-way social media posting activities that use these platforms for public service transactions, including call for action related to sharing a location or website to sign up for vaccination.

**Pull 1: citizen engagement**

- Refers to social media posting activities that aim to have a bidirectional engagement between public officials and the public. Interactions include authorities replying, retweeting posts by the public, mentioning accounts of the public, and relaying information from the public.

**Pull 2: public official engagement**

- Refers to social media posting activities that aim to have a bidirectional engagement among public officials. Interactions include authorities replying, retweeting posts by other authorities, mentioning, and quoting posts from other authorities.

Next, we described characteristics of tweets to guide our coding of sentiment (positive, negative, or neutral) and interaction type (Multimedia Appendix 5). Positive sentiment tweets use emojis, images, and adjectives that support vaccines and vaccine rollout; negative tweets express disagreement and anger toward the vaccine rollout; and neutral tweets are fact-based information with no emotional cues concerning vaccine rollout. Finally, we conducted a thematic analysis of the tweets to add details to our findings on sentiment, interaction type, and content.

### Ethical Considerations

Ethics approval was not required for this study as we conducted a secondary analysis of publicly available data.

## Results

### Overview

The analysis yielded 2 sets of findings. First, we present descriptive results derived from Brandwatch related to the volume of tweets from the 142 public officials accounts, either tweeted by these public officials or by others who have tagged, mentioned or replied to, or quoted these accounts. Next, we present a content analysis of the 270 included tweets, namely engagement (impressions, retweets, likes, and replies), sentiment (with examples of tweets), and interaction type (as described in Textbox 3). To enhance the findings from our content analysis, we present specific tweets that related to themes on sentiment and interaction type. For the 270 tweets coded, we calculated the interrater reliability between the 3 coders using Krippendorff α for nominal data, achieving an α coefficient of .811 for sentiment analysis and .784 for the content analysis of interactions (push-pull dynamics) of tweets, indicating very good and good agreement among coders, respectively.

### Description of Tweet Volume by Public Officials Across Phases and Within Included Provinces

**Figure 1:**
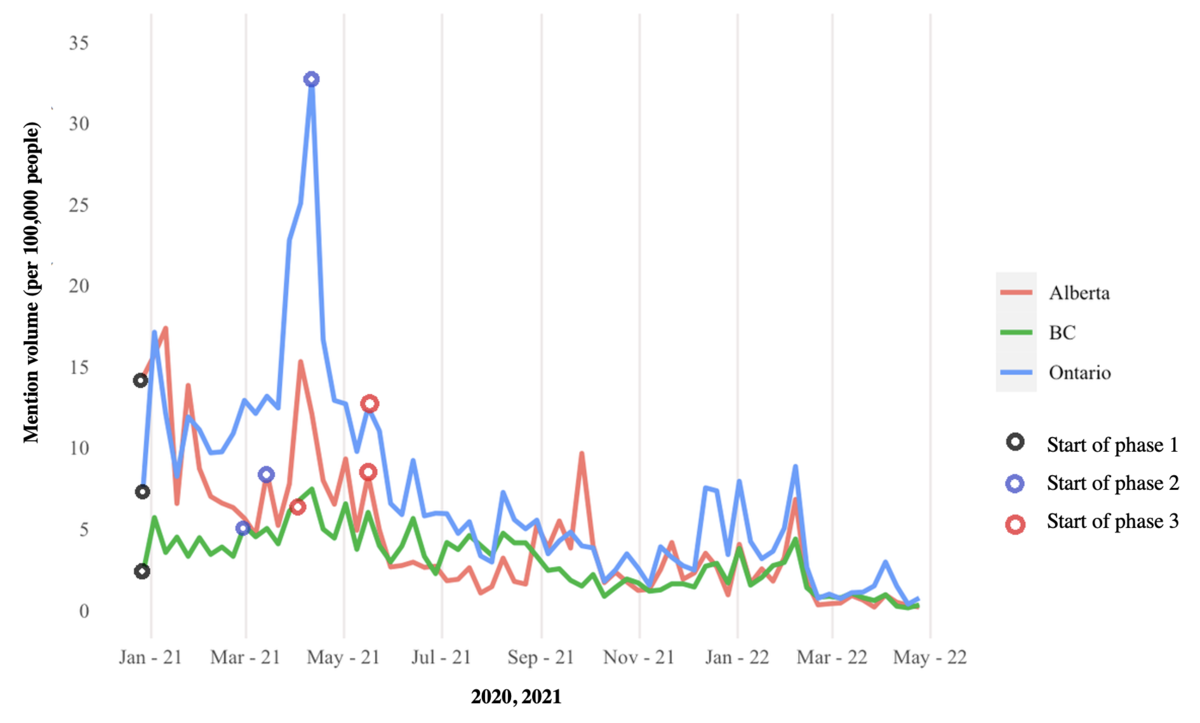
Mention volume of tweets from or to public officials during the 2020-2021 period, stratified by province. BC: British Columbia.

**Figure 2:**
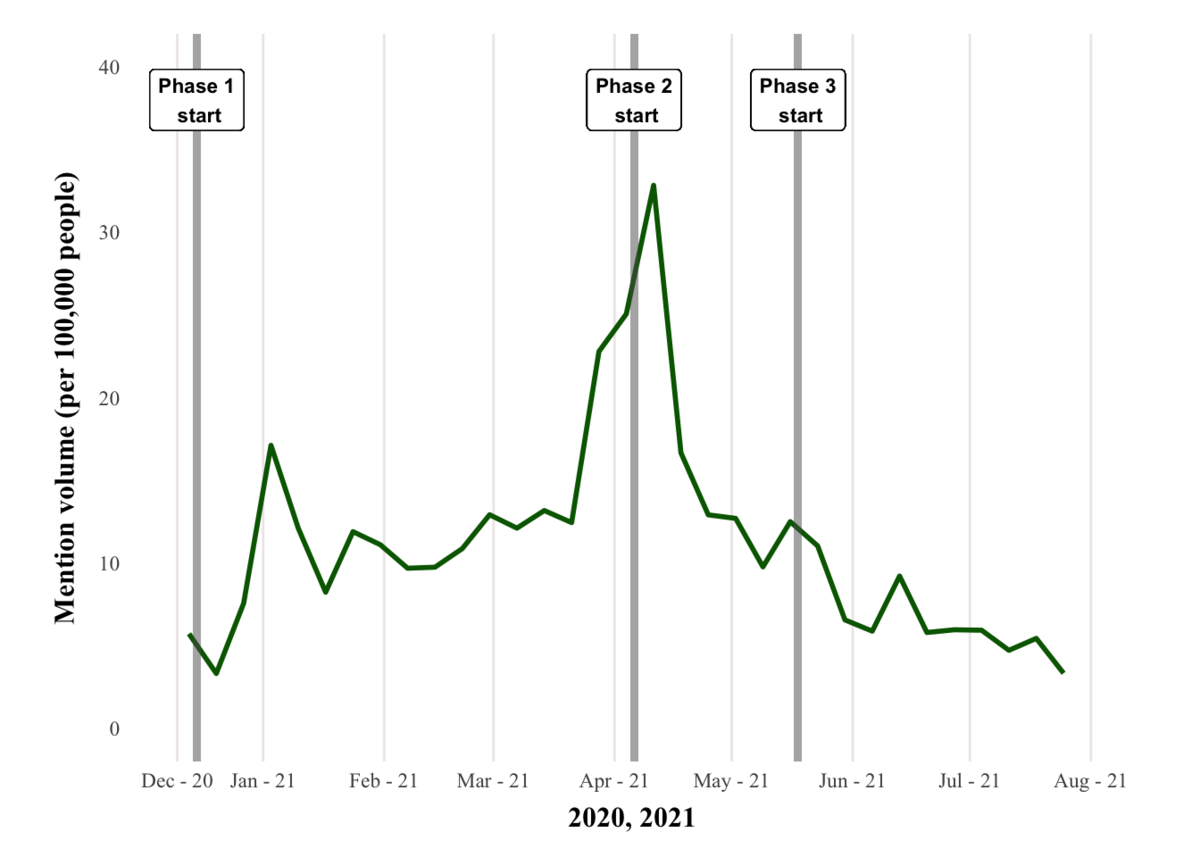
Mention volume of tweets from or to public officials in Ontario (7-day rolling average). Phase 1, phase 2, and phase 3 (gray lines) indicate the starting date of the vaccine rollout phase change.

**Figure 3:**
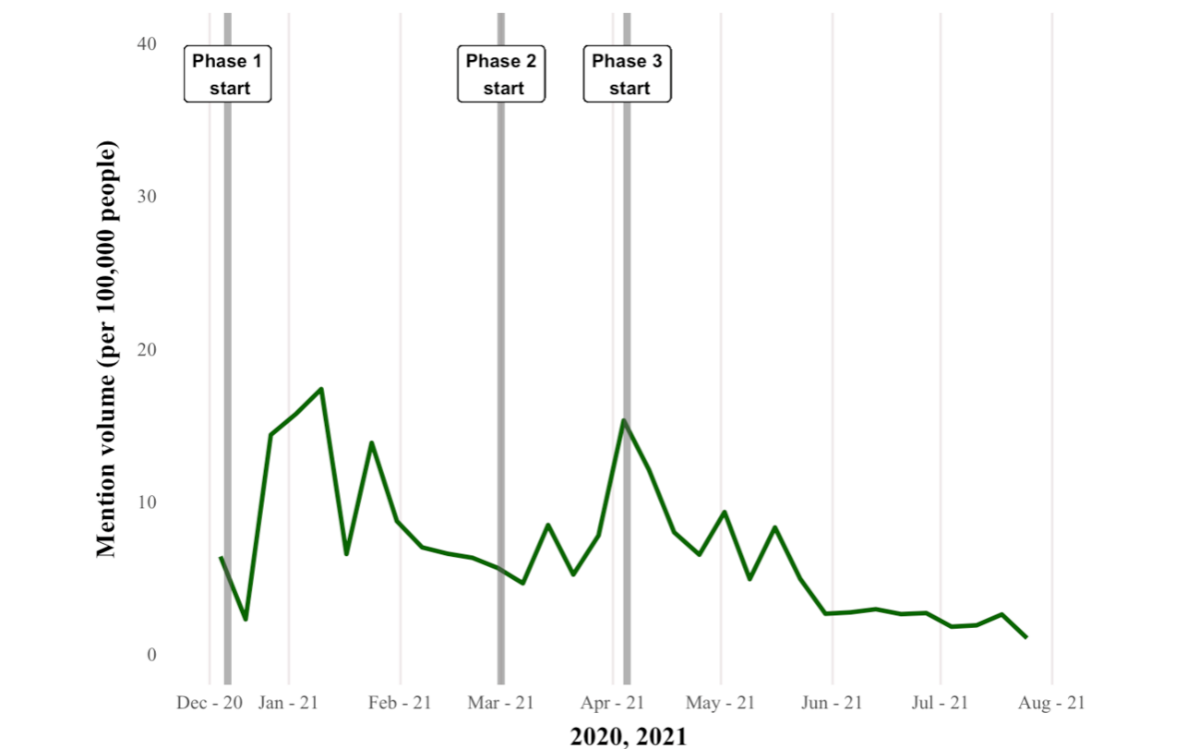
Mention volume of tweets from or to public officials in Alberta (7-day rolling average). Phase 1, phase 2, and phase 3 (gray lines) indicate the starting date of the vaccine rollout phase change.

**Figure 4:**
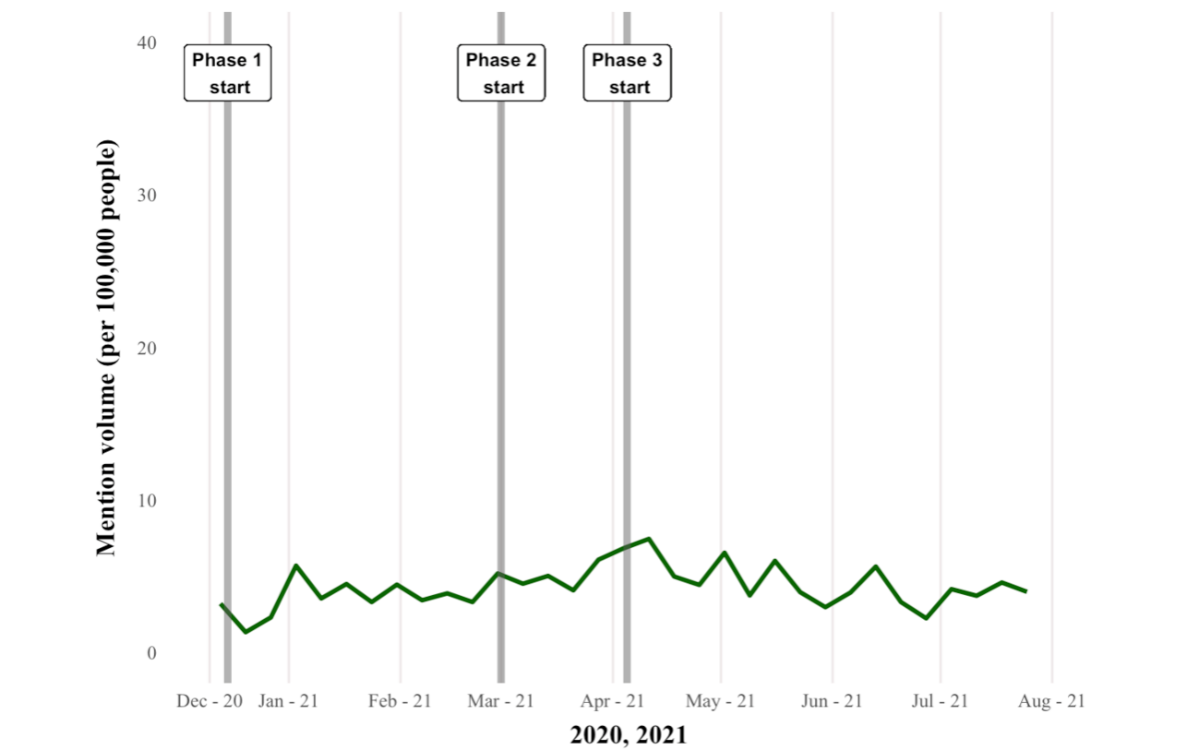
Mention volume of tweets from or to public officials in British Columbia (7-day rolling average). Phase 1, phase 2, and phase 3 (gray lines) indicate the starting date of the vaccine rollout phase change.

A total of 602,050 tweets from 153,200 unique Canadian users (identified by user geotags) were downloaded (Multimedia Appendix 6). During our extraction period, mention volumes were elevated from the end of December to mid-January 2021 (phase 1) across the 3 provinces (Figure 1). The second peak of conversation occurred at the beginning of phase 2 in April 2021 for Ontario (Figure 2) and Alberta (Figure 3), but similar patterns were not observed in BC (Figure 4). Ontario had the most mentions of public health officials (440,013 tweets by 53,431 unique users), BC had 53,472 tweets by 8653 unique users, and Alberta had 108,580 tweets by 13,274 unique users. Multimedia Appendix 7 provides the context for these phase changes, showing the number of vaccines administered in addition to the newly confirmed COVID-19 cases per day (*case positivity*) calculated by the 7-day moving average. The surge in tweet volume in Ontario in phase 2 (on March 15, 2021) coincided with the province’s third wave of COVID-19 transmission (Figure 1 and Multimedia Appendix 7). The highest volume of tweets in BC closely preceded BC’s phase 3 vaccine rollout and the province’s third wave of COVID-19 transmission. Finally, in Alberta, the highest volume of tweets matched the beginning of Alberta’s phase 2 vaccine rollout at the height of the province’s third wave. In contrast to the expectations, mention volume decreased to varying degrees in all provinces at phase 3, where all provinces witnessed peak COVID-19 transmission (Multimedia Appendix 7).

**Figure 5:**
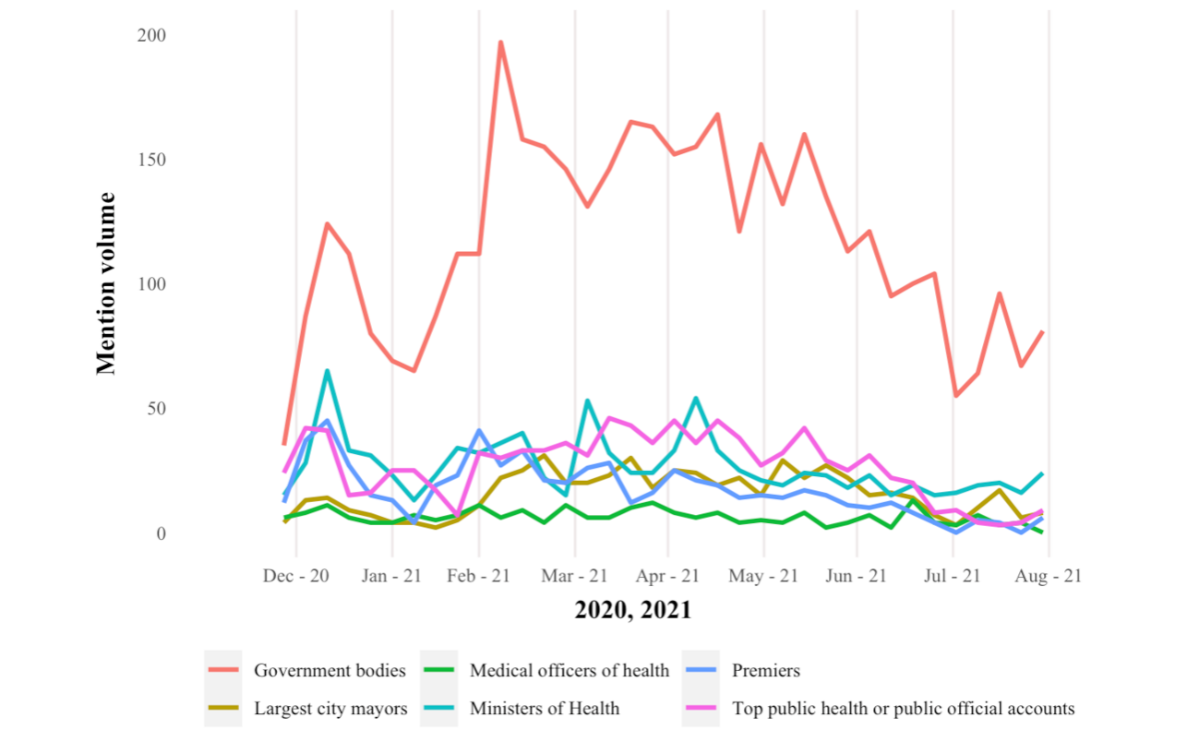
Mention volume of tweets from or to public officials during the 2020-2021 period, stratified by category of public official.

**Figure 6:**
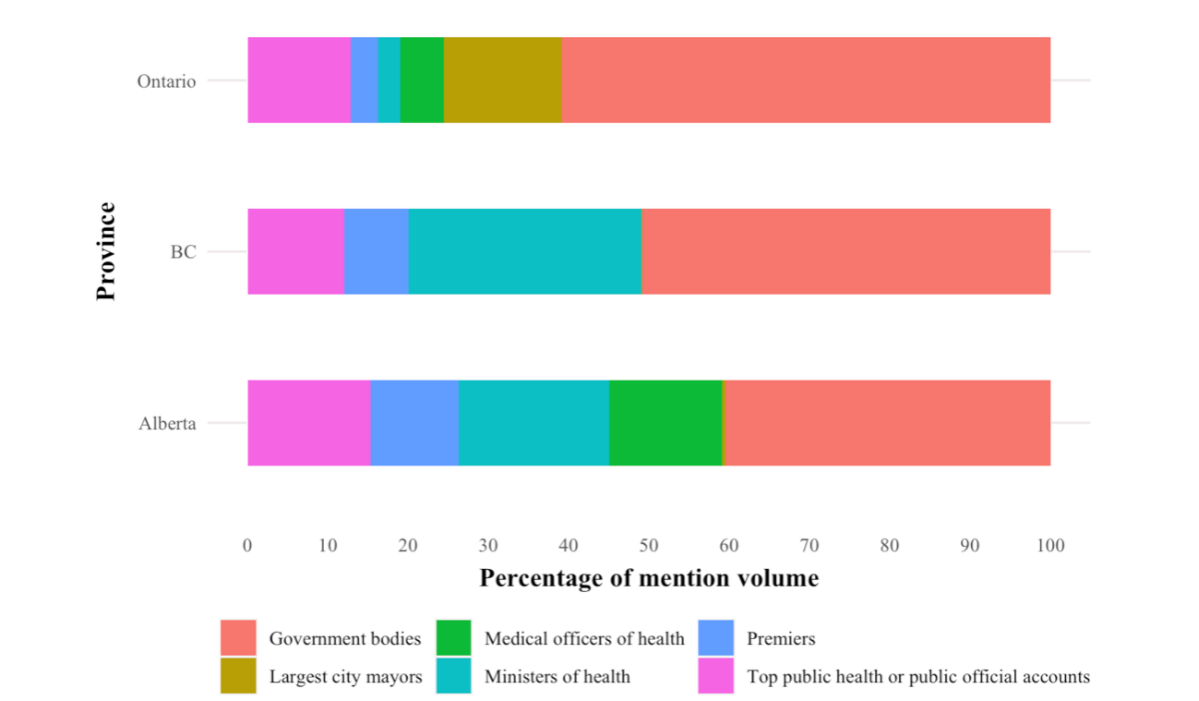
Relative percentage share of mention volume of tweets for each category of public official during the 2020-2021 period, stratified by province. BC: British Columbia.

Among the 142 public official accounts, government bodies (eg, regional health authorities, provincial government, and public health agencies; n=48) were the most prevalent in the vaccine rollout conversation. This pattern was observed in all 3 provinces and across all periods, though it was most evident at the start of phase 2 in Ontario (around March to April 2021) at the beginning of the third wave, during which the public first became eligible for the first dose of the COVID-19 vaccine (Figure 5). Within provinces, government officials had the highest proportion of the mention volume in Ontario (54/90, 60%), likely explained by the number of federal ministers in the data set who reside or tweet from Ottawa. Government officials are somewhat less represented in BC (45/90, 50%) and represent less than half (35/90, 39%) of the mention volume in Alberta. In Alberta and BC, Ministers of Health and first ministers (premiers) engaged more than their counterparts in Ontario (Figure 6).

### Content Analysis of Tweets With the Highest Impression

Multimedia Appendix 8 presents engagement metrics in Alberta, BC, and Ontario of tweets with the highest impression (viewership) in each province for each of the 3 phases of vaccine rollout (n=270). Tweets from public officials (212/270, 78.5%) and members of the public (25/270, 9.3%) and media (33/270, 12.2%) who retweeted, replied to, or mentioned public officials were included (definition provided in Multimedia Appendix 5). Table 1 presents the interaction types per our adapted public sector social media interaction framework (Textbox 3).

Table 2 presents a summary of themes for each interaction type. During the 26-day period around each of the 3 phases of rollout across Alberta, BC, and Ontario, 78.5% (212/270) of the most engaged public official tweets were tweeted by public officials, and 21.5% (58/270) of the tweets were from the media and the public who mentioned, quoted, and retweeted public officials. Information provision tweets (*push 1*) by public officials accounted for 65.6% (139/212) of the sampled tweets; public officials interacting with other public officials (37/212, 17.5%), or horizontal engagement (*pull 2*), were more common than public officials interacting with nonpublic officials, or vertical engagement (*pull* *1*; 24/212, 11.3%). The least common type of engagement was public service announcement tweets (*push 2*), which were only observed in Alberta (eg, tweets that promoted vaccine booking sites or where and how to claim vaccine passports), accounting for 5.7% (12/212) of the tweets sampled.

**Table 1:** Social media interaction across public officials for each province (n=212).

Interaction type	Alberta, n (%)	British Columbia, n (%)	Ontario, n (%)	Total^a^, n (%)
Push 1: information provision	33 (15.6)	63 (29.7)	43 (20.3)	139 (65.6)
Push 2: public service announcement	12 (5.7)	0 (0)	0 (0)	12 (5.7)
Pull 1: citizen engagement^b^	7 (3.3)	6 (2.8)	11 (5.2)	24 (11.3)
Pull 2: public official engagement	23 (10.8)	3 (1.4)	11 (5.2)	37 (17.4)

^a^The percentage of interaction of 212 included tweets by public officials.

^b^Only tweets *by* public officials were analyzed. Tweets engaged with public officials by the public and the media are not considered tweets by public officials.

**Table 2:** Summary of themes by the modified social media public service interaction framework.

Interaction type	Themes of tweets
Push 1: information provision	Tweets concerning provincial vaccine rollout policy: How many and what types of COVID-19 vaccines were received (phases 1 and 2) How many vaccines were administered (phases 1-3) Communicating to the public about who should receive the vaccine first; for example, frontline workers, older adults, immunocompromised, hot spots, susceptible populations, or underserved communities (phases 1-3) Celebratory tweets about vaccine rollout being on schedule (phase 3: April 2021 in British Columbia and May 2021 in Alberta and Ontario) Tweets concerning Federal vaccine rollout policy: Progress on vaccine distribution to Canada (phase 1) Progress on vaccine distribution to the Provinces (phase 2) Progress on vaccine administration in Canada (phase 3) Tweets concerning COVID-19 vaccine safety and efficacy for the Canadian population: Vaccine approval, safety, efficacy, and contraindications by age groups, gender, pregnancy status, and underlying medical conditions (phase 1) Updates on recommended dosages, interval, and booster requirements (phase 2 and 3)
Push 2: public service announcement	Provincial and municipal announcements and sharing of web-based vaccine booking sites, or rollout timeline changes with a corresponding URL to a newsletter subscription invitation (phases 1-3)
Pull 1: citizen engagement	Provincial public officials engaging with citizens; for example, by retweeting, replying, mentioning, and tagging contents and comments from the public concerning vaccine rollout (phase 2 and 3)
Pull 2: public official engagement	Provincial government and public health authorities retweeting updates posted by provincial ministers of health (phase 2 and 3) Federal ministers mentioning other federal provincial government and public health authorities (Federal to Federal) Members of the legislative assembly or provincial parliament (categorized as top public health officials or public officials) criticizing federal and provincial vaccine rollout (provincial to Federal, provincial, or other provinces)

Tweets categorized as information provision (*push 1*) in provinces were generally about the status on the arrival of vaccines, administration of vaccines (eg, number administered), and vaccine eligibility (eg, age and location) updates. Provincial and federal members of parliament (eg, Elizabeth May) with a large social following or influence were observed to retweet information provisional tweets, further driving the overall impression and reach of the original tweets by public officials.

Across the 3 provinces, public officials in Alberta had the highest prevalence of horizontal engagement (*pull 2*; Table 2). These *pull-2* interactions mainly involved praising or criticizing members of the cabinet responsible for the vaccine rollout. The highest prevalence of *pull-1* tweets was observed in Ontario, Canada. These interactions involved public officials mentioning, replying to, or quoting members of the public and the media. This interaction between public officials and influential nonpublic officials (>1000 Twitter followers) generated high viewership and were better endorsed (described in the *Sentiment Analysis* section) than *push-1* and *push-2* type tweets, such as those who tagged the Minister of Health offering to volunteer as vaccinators; tweets from journalists using their personal accounts to broadcast the latest government decisions on vaccine procurement, delivery, and eligibility; and express opinions on issues pertinent to the phase changes. We did not observe the same phenomenon for tweets with high impressions in BC or Alberta.

### Sentiment Analysis

**Figure 7:**
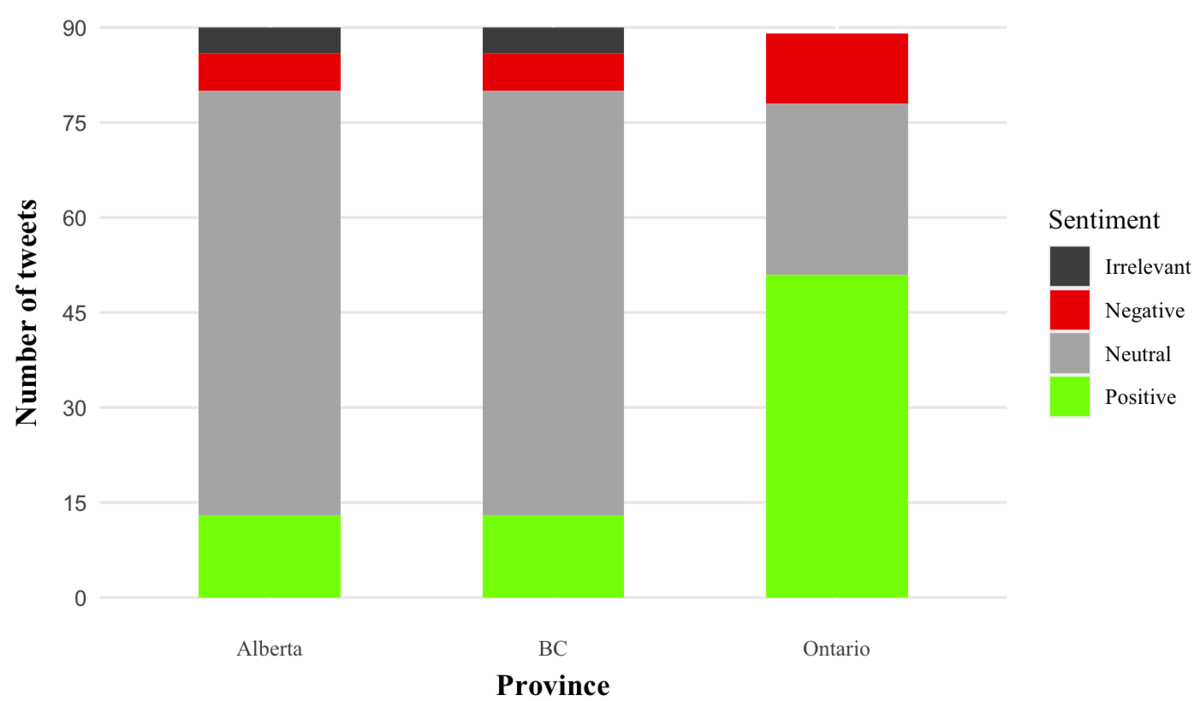
Sentiment of extracted tweets during the 2020-2021 period, stratified by province. BC: British Columbia.

Across the provinces, most tweets (138/270, 51.1%) conveyed neutral sentiment across all phases. In BC, 74% (67/90) of the public officials’ tweets were neutral, followed by 48% (43/90) in Alberta, and 31% (28/90) in Ontario, likely attributable, in part, to the abundance of *push*-*1* type tweets that focused on information provision. Negative sentiment accounted for 12.6% (34/270) of all the tweets. Remaining tweets conveyed positive sentiment; Ontario had the highest proportion of positive sentiment (54/90, 60%), followed by Alberta (30/90, 33%) and BC (13/90, 14%; Figure 7). Positive sentiment is demonstrated by public officials to invoke the public for their ongoing commitment to get vaccinated. In Ontario, for example, the Mayor of Toronto expressed gratitude to frontline HCWs for receiving their vaccination, whereas other public officials used a positive tone to thank the public for collaborating with vaccination efforts during the pandemic. In addition, public officials invoked other arms-length provincial government organizations and public health authorities (eg, Canadian Armed Forces) for helping to procure and distribute vaccines, particularly in hard-to-reach or priority areas (Northern Canada and residential long-term care facilities), as well as HCWs and facilities, including physicians and pharmacies. Often, such tweets were associated with dynamic and compelling images with smiling frontline workers, emojis that convey excitement, the use of exclamation points, and a positive overall tone of the tweet (Multimedia Appendix 5). For example, in BC, physicians and executives in the health system praised and expressed positive sentiment toward public officials and quoted tweets from public official accounts to endorse vaccine efforts in phase 1 (December 2020). More examples of positive and negative sentiment tweets are shown in Multimedia Appendix 9.

Across all provinces, negative sentiment (Ontario: 6/90, 7%; Alberta: 17/90, 19%; BC: 12/90, 13%) was tied to feelings of anger and frustration toward federal and provincial public officials for not meeting stated vaccine rollout goals. For example, members of the public in Alberta and Ontario invoked provincial public officials in criticisms around vaccine rollout. Out of the sampled tweets and their responses, no public officials responded to these criticisms directly.

In Multimedia Appendix 8, endorsement ratios (numbers of likes/number of impressions) indicate how receptive Twitter users are toward the vaccine rollout tweet posted by a certain user; the higher likes or impressions, the better endorsed a tweet is. Across the 3 provinces, tweets by and to public officials received a median endorsement ratio of 0.0002. Comparing between provinces, First Ministers in Ontario had the highest endorsement ratio (0.0009) compared with their counterparts in BC (0.0002) and Alberta (0.0007). The low endorsement ratio in BC can be explained by BC’s First Minister only tweeting one popular tweet during the peaks of provincial vaccine rollout.

The most popular Minister of Health across the 3 provinces is BC’s Adrian Dix (endorsement ratio: 0.0008). In terms of Chief Medical Officers of Health (CMOH), Alberta’s CMOH tweeted consistently (18/90, 20%) and received a higher endorsement ratio (0.0006) compared with the CMOH in Ontario (1/90, 1%; 0.0002) and BC (n=0), as BC’s CMOH did not have a Twitter presence. Albertan public officials engaged in all 4 methods of engagement (ie, push 1, push 2, pull 1, and pull 2). In Ontario, 3 methods of engagement were used. In BC, 2 methods of engagement were used. Across categories, government bodies in Alberta received the highest endorsement ratio across all provinces (Alberta: 0.0007, BC: 0.0001, and Ontario: 0.0002). The most active municipal officials were from Ontario (23/90, 26%; endorsement ratio=0.0002), who had the highest endorsement ratio compared with BC (n=0; 0.0000) and Alberta (1/90, 1%; 0.0001).

Media endorsement across the provinces was low in Alberta and BC, indicating large viewership and low engagement (Alberta: 0.0000 and BC: 0.0000), whereas tweets from the public received higher endorsement (Alberta: 0.0025 and BC: 0.118). This observation contrasts with Ontario (19/90, 21%; 0.0004), where the media received higher endorsement ratios than tweets from the public (7/90, 8%; 0.0032).

## Discussion

### Principal Findings

We note 2 salient findings from our results concerning the use of Twitter by public officials to communicate about COVID-19 vaccine rollout in Canada. First, out of all 10 provinces, public officials in 3 provinces—Alberta, BC, and Ontario—use Twitter the most. Out of all 142 sampled public officials’ accounts, Twitter was mainly used for unidirectional information provision (*push 1*) to update the public on numbers of vaccines administered. In Ontario and Alberta, we observed a pattern around tweet volume and phase of rollout. An increase in public official interactions on Twitter coincided with the onset of the third wave of the COVID-19 pandemic and matched the start of phase 2 vaccine rollout (phase 2 in Alberta and Ontario: March to April 2021). This can be explained by the governments’ readiness to expand vaccine eligibility after the vaccine shortages were resolved. BC, in contrast, had a relatively steady volume of social media interactions by and to public officials across all account categories, which can be explained by the relatively lower daily cases recorded and the absence of public officials from BC on Twitter compared with Ontario and Alberta. BC’s CMOH is not on Twitter, which could help explain the lack of any discernable patterns around the use of other interaction types beyond information provision in this province. Social media presence among public officials as a determinant of engagement is therefore a unique area of future investigation. In contrast to our expectations, we also observed a decrease in mention volume across all provinces at phase 3, despite this phase coinciding with peak COVID-19 transmission. We surmise that other COVID-19 conversations overtook vaccine discourse in the public domain by this point.

Second, out of the top viewed tweets, much of the information provided about vaccination rollout on Twitter came from 2 categories of public officials: government bodies (including public health authorities at the federal and provincial levels) and the largest city mayors. Despite accounting for the highest mention volume of tweets, which we attributed to their overrepresentation in our sample, government bodies yielded the lowest endorsement ratios (based on likes and impressions) across all provinces. In comparison, mayors who embedded images and animations in tweets expressing appreciation for frontline workers and the public’s vaccination efforts received greater endorsement. This observation is supported by another Canadian study that showed that accounts that tweet frequently per day experience lower engagement per tweet, especially when those tweets do not involve hashtags or multimedia such as animated *gifs* or videos [36]. In contrast, across all 3 provinces, popular tweets by other key public officials not directly responsible for vaccine rollout across received higher than average endorsement, likely attributable to presenting views endorsed by the public (eg, voicing concerns about, or praising, vaccine rollout). Accordingly, we note an opportunity for public officials to engage with other public officials (*pull 2*) to explore bidirectional engagement and its effect on public endorsement during crisis communication.

Furthermore, an interplay of factors explains why a tweet receives many views and many likes (thereby resulting in a high endorsement ratio). For example, the reader may agree with the tweet or show support for the tweet [37]. This was observed during the initial phases of the COVID-19 pandemic when the public liked the tweets of National Health Service to express gratitude [37]. In addition, reading tweets from familiar celebrities correlated with higher endorsement of vaccinations according to a nationwide Twitter experiment that recruited celebrities to endorse vaccination on Twitter [38]. Similarly, there can be many reasons for a tweet to receive many views but a low volume of likes: as impressions are presented as one opens their Twitter feed, tweets that are paid promotions without images or links, such as social marketing campaigns to promote COVID-19 vaccine–related services and information, will likely result in a low endorsement ratio [39]. In addition, viewers may not *like* a tweet when the tweeted content does not align with their beliefs (eg, vaccine beliefs or vaccine eligibility criteria). In a recent large-scale Twitter study, there was substantial empirical evidence pointing toward Twitter’s algorithmic amplification of politically right-winged beliefs in Canada [40]; these right-wing beliefs tend to correlate with weaker COVID-19 risk perceptions [41].

Regarding the differences in the sentiment of public officials among the 3 provinces, higher positive sentiment can be explained by the high prevalence of government bodies and mayoral accounts that drove the most views. In particular, Ontario’s positive sentiment came from a disproportionately high percentage of government bodies and largest city mayors who used affirming words (eg, “great” and “thanks”), emotive punctuation (eg, exclamation points) to emphasize excitement toward the public’s uptake of the COVID-19 vaccine, and emojis implying celebration and strength in numbers (eg, clapping hands and flexing arms). Our findings on positive emoji use echo recent scholarship that notes emoji use by the public during COVID-19 overwhelmingly conveyed positive sentiment [42]. The findings in Ontario on social media behavior driving positive sentiment contrast with Alberta and BC, where government bodies used neutral words and expressions and did not use emojis when conveying information about vaccines and engaging with citizens.

As information about tweet sponsorship by public officials for social marketing campaigns is not publicly available, it is inconclusive why specific public officials’ tweets are endorsed more than others. Regardless, it has been noted that highly endorsed tweets are correlated with perceived credibility, which in turn draws more likes [43]. In Ontario, that the highest endorsement ratio was observed for tweets by the Premier may suggest that tweets about vaccine rollout from the Premier, a controversial figure with right-leaning ideology, are well liked. This finding may be surprising to those with opposing ideologies who have been critical of the Premier’s pandemic response; however, this may reflect a phenomenon known as *majority illusion* on social media, which suggests that the opinion of a few, amplified in respective echo chambers on social media for which we perceive as a dominant opinion, may in fact be the minority opinion [44].

### Comparison With Prior Work

Effective engagement with the public over social media is critical during times of crisis, particularly to protect public safety; to maintain open, clear, and transparent communication of complex issues and risk calculations; and to maintain support for ongoing public health measures and trust in governments. Indeed, recent scholarship from Canada notes the importance of communication strategies by all orders of government to shape change during the COVID-19 pandemic. In particular, transparency is critical in sustaining public trust [45]. Our study found that public officials did promote transparency through the use of Twitter to provide information to the public during the initial rollout of the COVID-19 vaccine, but there was limited engagement and dialogue with the public during this time. Previous studies suggest that public officials at local levels of government have the closest connection to the public, but, as we also found, have used social media in an unengaging and fragmented manner [46-48]. Earlier work has shown a general reluctance by government officials to use social media to engage with the public in times of crisis [49], although this appears to be changing during COVID-19 as noted in our study and elsewhere.

Given the important role of social media communication by government officials in times of crisis, evidence is emerging regarding the use of Twitter by public officials during the COVID-19 pandemic. Zeemering [50] described fragmented communication across municipal public sectors in 3 states in the United States in the early months of the COVID-19 pandemic. They noted that mitigating challenges in communicating public health messaging about COVID-19 requires coordination across all public sectors to ensure better amplification about pandemic responses. In our findings on vaccine communication, this fragmentation was not evident, in part because we focused on multiple layers of government providing uncoordinated messages. Our research focused on communication about vaccine rollout specifically, which was largely the responsibility of government bodies (eg, provincial government and public health authorities), a first minister (health), or a CMOH for provincial updates, and local mayors for municipal updates.

Our findings are consistent with previous research that observes that information provision is the most common type of interaction on social media by public officials [47]. Interestingly, this is inconsistent with a recent study from Poland that also categorized social media communication by public officials during the COVID-19 pandemic using the framework by Mergel [11]. Their analysis of Facebook, Instagram, and Twitter use during the pandemic by local government officials suggests pushing information to be the least used type of interaction. Further work could explore whether public officials in other jurisdictions and at different levels of government use social media in different ways.

### Limitations

This study has several limitations. From a methodological perspective, we analyzed a small sample of tweets across select provinces to inform Brandwatch’s social media intelligence platform and categorize and track public officials’ engagement activities across larger geographic contexts (eg, all Canadian provinces and territories). Along with limited resources and this serving as a pilot study, we were limited in the volume of sentiment and content analysis that we could perform. Via Brandwatch’s retrospective database, there were no means to differentiate whether a tweet was promoted (paid) to generate more views and audiences [51], and only geotagged tweets were included.

From an analytic perspective, our study focuses on public officials’ engagement on Twitter around the vaccine rollout. Accordingly, we do not analyze other popular social media platforms that may target different audiences, such as Facebook and Instagram, which have been studied in the context of government engagement during crises such as COVID-19 in other countries [11,52,53]. It is possible that several elected public officials do not use Twitter, have Twitter but are inactive, are represented by an organizational user account, or do not have a substantive or engaged following, but are highly engaged on other social media platforms [36]. Furthermore, given the small sample of tweets from which we extracted content and the labor-intensive process of manually coding engagement types (per the public sector social media interaction framework by Mergel [4]), we could not compare how engagement changed or remained consistent across phases of vaccine rollout within each province. In addition, our measures of engagement did not account for the public officials’ follower count. Finally, we did not look at the impact of the different types of users or interaction types on vaccine uptake, which could be a focus of future research. To narrow the scope of this study, we did not analyze public resonance to public officials’ tweets, but this represents another focus of future research.

### Conclusions

The COVID-19 pandemic is an objective lesson in the importance of communicating timely information about vaccine availability to reduce COVID-19 spread. Findings from our study conducted through a Canadian lens advance a growing body of literature on how public officials use social media, particularly Twitter, to communicate with the public during the COVID-19 pandemic. We found a predominant use of information provision (*push-1* interaction) and a reliance on official government accounts to communicate information, which may not be as effective at engaging the public. Our findings leave room for further research, particularly around developing a set of best practices that public officials can lean into when developing communication strategies in times of crisis, COVID-19 related or otherwise.

## Appendix

Multimedia Appendix 1 Number of public officials included in each jurisdiction (federal, provincial, or territorial).

Multimedia Appendix 2 Full query.

Multimedia Appendix 3 Vaccine rollout phase changes and milestones in Alberta, British Columbia (BC), and Ontario for the first and second doses.

Multimedia Appendix 4 Data collection flowchart.

Multimedia Appendix 5 Definitions of variables extracted for 270 tweets.

Multimedia Appendix 6 Flow diagram of tweet selection.

Multimedia Appendix 7 COVID-19 vaccine administration and case positivity in Alberta, British Columbia, and Ontario (December 28, 2020, to August 31, 2021).

Multimedia Appendix 8 Public officials’ Twitter engagement metrics.

Multimedia Appendix 9 Examples of tweets conveying positive and negative sentiment.

## Abbreviations

BC: British Columbia
CMOH: Chief Medical Officers of Health
HCW: health care worker

## Data Availability

Elements of the data sets generated and analyzed during this study are not publicly available because of the proprietary nature of the analytic services provided by Brandwatch Analytics. A data set that has been stripped of the proprietary information will be made available upon reasonable request from the corresponding author.
