# Supplementary material for: Public Officials’ Engagement on Social Media During the Rollout of the COVID-19 Vaccine: Content Analysis of Tweets
Source: JMIR Infodemiology. 2023 Jul 20;3:e41582. doi: 10.2196/41582 (PMC10361259; doi:10.2196/41582)
Supplement: Multimedia Appendix 1 [file infodemiology_v3i1e41582_app1.docx]

Multimedia Appendix 1. Number of public officials included in each jurisdiction (Federal, Provincial, Territorial)

| **Jurisdiction** | **Categories of Public Officials** | | | | | | | |
| --- | --- | --- | --- | --- | --- | --- | --- | --- |
|  | **1. Premier** | **2. Minister of Health** | **3. Chief Medical Officer** | **4. Government Bodies** | **5. Capital City Mayors** | **6. Top public health/public official accounts** | **Total** | |
| **Federal** | 2 | 1 | 1 | 4 | 1 | 13 | 22 | |
| **BC**^c^ | 1 | 1 | 0^a^ | 7 | 1 | 0 | 10^b^ | |
| **Alberta**^c^ | 1 | 2 | 1 | 2 | 1 | 3 | 10 | |
| **Manitoba** | 1 | 2 | 1 | 7 | 1 | 1 | 13 | |
| **New Brunswick** | 1 | 1 | 0^a^ | 5 | 1 | 2 | 10 | |
| **Nova Scotia** | 1 | 1 | 0^a^ | 4 | 1 | 4 | 11 | |
| **Prince Edward Island** | 1 | 1 | 0^a^ | 3 | 1 | 2 | 8 | |
| **Newfoundland** | 1 | 1 | 1 | 6 | 1 | 2 | 12 | |
| **Northwest Territories** | 1 | 1 | 1 | 2 | 1 | 1 | 7 | |
| **Yukon** | 1 | 1 | 0^a^ | 2 | 1 | 1 | 6 | |
| **Nunavut** | 1 | 0^a^ | 0^a^ | 1 | 1 | 1 | 4 | |
| **Saskatchewan** | 1 | 2 | 0^a^ | 2 | 1 | 3 | 9 | |
| **Quebec** | 1 | 1 | 1 | 4 | 2 | 2 | 11 | |
| **Ontario**^c^ | 1 | 1 | 0^a^ | 4 | 1 | 3 | 10 | |
| **TOTAL** | 15 | 16 | 6 | 53 | 14 | 38 | 142 | |
| ^a^ Respective public official exists but does not have Twitter account  ^b^ No additional public officials derived after secondary search  ^c^ The three provinces with the highest volume of vaccine rollout tweets from/to public officials | | | | | | |  |  |
|  |  |  |  |  |  |  |  |  |
