# Supplementary material for: Public Officials’ Engagement on Social Media During the Rollout of the COVID-19 Vaccine: Content Analysis of Tweets
Source: JMIR Infodemiology. 2023 Jul 20;3:e41582. doi: 10.2196/41582 (PMC10361259; doi:10.2196/41582)
Supplement: Multimedia Appendix 2 [file infodemiology_v3i1e41582_app2.docx]

Multimedia Appendix 2. Full query

| (((vaccin* OR vax* OR immuniz* OR immunis*) AND (distribu* OR allocat* OR roll-out OR "roll out" OR deliver* OR provid* OR provision OR administer* OR administr* OR livraison OR Apporter OR alloue*)))  AND  (((@CMOH_Alberta OR @roussin_brent OR @CMOH_NL OR @NWT_CPHO OR @ArrudaHoracio OR @CPHO_Canada OR @kennedystewart OR @nenshi OR @Mayor_Bowman OR @MayorMoncton OR @MikeSavageHFX OR @PublicHealthCZ OR @PhilipBrownPEI OR @DannyBreenNL OR @DanCurtisYXY OR @MayorofIqaluit OR @charlieclarkyxe OR @Santepub_Mtl OR @Val_Plante OR @JohnTory OR @epdevilla OR @AnitaOakville OR @BCGovNews OR @PHSAofBC OR @VCHhealthcare OR @Fraserhealth OR @Interior_Health OR @Northern_Health OR @VanIslandHealth OR @YourAlberta OR @AHS_media OR @MBGov OR @WinnipegRHA OR @SharedHealthMB OR @NorthHealthMB OR @PrairieMtHealth OR @IERHA_MB OR @SouthernSante OR @Gov_NB OR @vitalitenb OR @HorizonHealthNB OR @nsgov OR @HealthNS OR @infopei OR @GovNL OR @HCS_GovNL OR @yukongov OR @GovofNunavut OR @SKGov OR @saskhealth OR @santemontreal OR @gouvqc OR @sante_qc OR @TOPublicHealth OR @ONThealth OR @ottawahealth OR @ONgov OR @canada OR @GovCanHealth OR @premierbhiggs OR @dennyking OR @Premier_Silver OR @Jsavikataaq OR @CCochrane_NWT OR @jjhorgan OR @jkenney OR @PremierScottMoe OR @brianpallister OR @fordnation OR @celliottability OR @francoislegault OR @FureyAndrew OR @IainTRankin OR @cafreeland OR @adriandix OR @shandro OR @jasonluan88 OR @mlastefanson OR @AudreyGordonMB OR @shepharddorothy OR @zachchurchill OR @erniehudsonPEI OR @Johnrockdoc OR @juliegreenmla OR @pfrostoldcrow OR @merrimanpaul OR @everetthindley OR @cdube_sante OR @PattyHajdu OR @adriandix OR @shandro OR @jasonluan88 OR @mlastefanson OR @AudreyGordonMB OR @shepharddorothy OR @zachchurchill OR @erniehudsonPEI OR @Johnrockdoc OR @juliegreenmla OR @pfrostoldcrow OR @merrimanpaul OR @everetthindley OR @cdube_sante OR @PattyHajdu OR @Gouv_NB OR @CityofMoncton OR @nshealth OR @PublicHealthCZ OR @Health_PEI OR @PEIwellness OR @EasternHealthNL OR @WesternHealthNL OR @CentralHealthNL OR @LGHealthNL OR @ACSP_TNO OR @NTHSSA OR @yukonhss OR @INSPQ OR @GovCanNorth OR @JimWatsonOttawa OR @pancholi_rakhi OR @RachelNotley OR @Drew__Barnes OR @CentreInfection OR @JohnWilliamson_ OR @DominicCardy OR @BernJordanMP OR @AndyFillmoreHFX OR @WayeMason OR @TimHoustonNS OR @hannahbethbell OR @karlabernardmla OR @SiobhanCoadyNL OR @twpiggott OR @RylundJohnson OR @nils_clarke OR @Maqaiti OR @AleanaYoung OR @Vicki_Mowat_NDP OR @WotherspoonT OR @drgbarrette OR @PierreArcand OR @AndreaHorwath OR @Sflecce OR @epdevilla OR @pablorodriguez OR @jyduclos OR @trudel_denis OR @PierrePaulHus OR @erinotoole OR @HedyFry OR @HarjitSajjan OR @ElizabethMay OR @MichelleRempel OR @RaquelDancho OR @stbstvdan OR @YvonneJJones OR @JustinTrudeau) AND country:can)  OR author:(CMOH_Alberta OR roussin_brent OR CMOH_NL OR NWT_CPHO OR ArrudaHoracio OR CPHO_Canada OR kennedystewart OR nenshi OR Mayor_Bowman OR MayorMoncton OR MikeSavageHFX OR PublicHealthCZ OR PhilipBrownPEI OR DannyBreenNL OR DanCurtisYXY OR MayorofIqaluit OR charlieclarkyxe OR Santepub_Mtl OR Val_Plante OR JohnTory OR epdevilla OR AnitaOakville OR BCGovNews OR PHSAofBC OR VCHhealthcare OR Fraserhealth OR Interior_Health OR Northern_Health OR VanIslandHealth OR YourAlberta OR AHS_media OR MBGov OR WinnipegRHA OR SharedHealthMB OR NorthHealthMB OR PrairieMtHealth OR IERHA_MB OR SouthernSante OR Gov_NB OR vitalitenb OR HorizonHealthNB OR nsgov OR HealthNS OR infopei OR GovNL OR HCS_GovNL OR yukongov OR GovofNunavut OR SKGov OR saskhealth OR santemontreal OR gouvqc OR sante_qc OR TOPublicHealth OR ONThealth OR ottawahealth OR ONgov OR canada OR GovCanHealth OR premierbhiggs OR dennyking OR Premier_Silver OR Jsavikataaq OR CCochrane_NWT OR jjhorgan OR jkenney OR PremierScottMoe OR brianpallister OR fordnation OR celliottability OR francoislegault OR FureyAndrew OR IainTRankin OR cafreeland OR adriandix OR shandro OR jasonluan88 OR mlastefanson OR AudreyGordonMB OR shepharddorothy OR zachchurchill OR erniehudsonPEI OR Johnrockdoc OR juliegreenmla OR pfrostoldcrow OR merrimanpaul OR everetthindley OR cdube_sante OR PattyHajdu OR Gouv_NB OR CityofMoncton OR nshealth OR PublicHealthCZ OR Health_PEI OR PEIwellness OR EasternHealthNL OR WesternHealthNL OR CentralHealthNL OR LGHealthNL OR ACSP_TNO OR NTHSSA OR yukonhss OR INSPQ OR GovCanNorth OR JimWatsonOttawa OR pancholi_rakhi OR RachelNotley OR Drew__Barnes OR CentreInfection OR JohnWilliamson_ OR DominicCardy OR BernJordanMP OR AndyFillmoreHFX OR WayeMason OR TimHoustonNS OR hannahbethbell OR karlabernardmla OR SiobhanCoadyNL OR twpiggott OR RylundJohnson OR nils_clarke OR Maqaiti OR AleanaYoung OR Vicki_Mowat_NDP OR WotherspoonT OR drgbarrette OR PierreArcand OR AndreaHorwath OR Sflecce OR epdevilla OR pablorodriguez OR jyduclos OR trudel_denis OR PierrePaulHus OR erinotoole OR HedyFry OR HarjitSajjan OR ElizabethMay OR MichelleRempel OR RaquelDancho OR stbstvdan OR YvonneJJones OR JustinTrudeau)) NOT (bovine OR cancer OR dog OR dengue OR ebola OR flu OR hepatitis OR HPV OR bird OR TB) |
| --- |
