# Supplementary material for: Public Officials’ Engagement on Social Media During the Rollout of the COVID-19 Vaccine: Content Analysis of Tweets
Source: JMIR Infodemiology. 2023 Jul 20;3:e41582. doi: 10.2196/41582 (PMC10361259; doi:10.2196/41582)
Supplement: Multimedia Appendix 3 [file infodemiology_v3i1e41582_app3.docx]

Multimedia Appendix 3. Vaccine rollout phase changes and milestones in Alberta, BC, and Ontario for the first and start of second doses. Intervention category: Vaccine rollout [36]

| **Province** | **Vaccine rollout phase changes and milestones** | **Start date** |
| --- | --- | --- |
| **Alberta** | Phase 1: Healthcare worker and long-term care home (1^st^ dose) | 15-Dec-20 |
|  | Phase 2A*: Ages 18+ and children born before 2005 with high-risk conditions | 15-Mar-21 |
|  | Phase 3: Ages 12 - 17 (1^st^ dose) | 10-May-21 |
|  | Start of 2^nd^ dose | 18-Jun-21 |
| **British Columbia** | Phase 1: Healthcare worker (1^st^ dose) | 15-Dec-20 |
|  | Phase 2: High risk and elderly populations (1^st^ dose) | 1-Mar-21 |
|  | Phase 3: Ages 18+ and children 12-17 (1^st^ dose) | 5-Apr-21 |
|  | Start of 2^nd^ dose | 4-Jun-21 |
| **Ontario** | Phase 1: Healthcare worker and long-term care home (1^st^ dose) | 15-Dec-20 |
|  | Phase 2: General population ages 50+ and COVID-19 hotspots (1^st^ dose) | 6-Apr-21 |
|  | Phase 3: Ages 18+ and children 12-17 (1^st^ dose) | 18-May-21 |
|  | Start of 2^nd^ dose | 24-May-21 |
| * In Alberta, phase 2 consisted of two stages (2A for those aged 18+ beginning on March 15, 2021, and 2B for those born before 2005 with high-risk conditions. We use the start of phase 2A as the initial date of analysis. | | |

Source: CIHI COVID-19 Intervention Timeline in Canada [36]
