# Supplementary figures and images for: Public Officials’ Engagement on Social Media During the Rollout of the COVID-19 Vaccine: Content Analysis of Tweets
Source: JMIR Infodemiology. 2023 Jul 20;3:e41582. doi: 10.2196/41582 (PMC10361259; doi:10.2196/41582)

Multimedia Appendix 4. Data collection flowchart


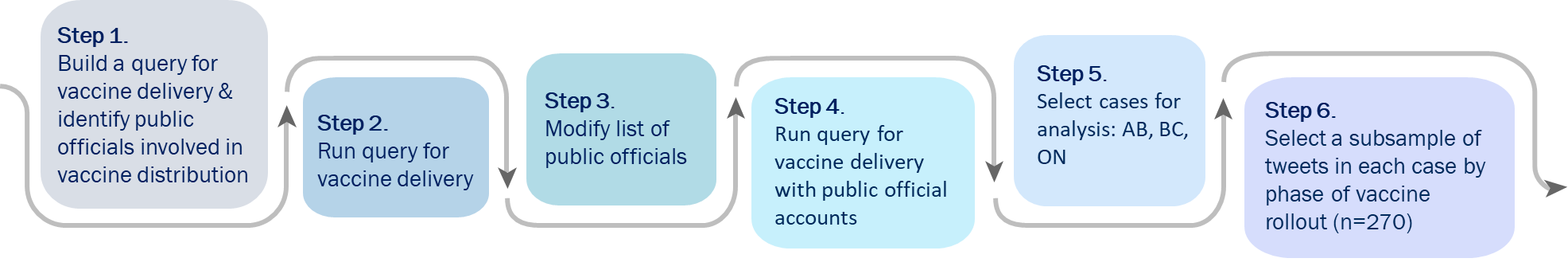

Supplement: Multimedia Appendix 4 [file infodemiology_v3i1e41582_app4.docx]
