# Supplementary material for: Public Officials’ Engagement on Social Media During the Rollout of the COVID-19 Vaccine: Content Analysis of Tweets
Source: JMIR Infodemiology. 2023 Jul 20;3:e41582. doi: 10.2196/41582 (PMC10361259; doi:10.2196/41582)
Supplement: Multimedia Appendix 5 [file infodemiology_v3i1e41582_app5.docx]

# Multimedia Appendix 5. Definitions of variables extracted for 270 tweets

| **Extracted information from tweets (variables)** | **Definition** |
| --- | --- |
| Author | Self-prescribed title and name of the Twitter account |
| Extraction Category | 1. Premiers or Prime Minister: Canadian elected provincial leaders and Federal leaders belonging to any political party. 2. Ministers of Health: Appointed provincial, federal, or territorial ministers of health in Canada, there is one Minister of Health in each Province and one Minister of Health at the Federal level. 3. Medical Officers of Health (MOH): Appointed provincial or federal physicians who are responsible for leading the public health system in its jurisdiction. 4. Government bodies: official organizational accounts of the Federal Government (i.e., Government of Canada); federal public health authorities (i.e., Health Canada and Public Health Agency of Canada); provincial governments (i.e., Government of New Brunswick), and provincial public health authorities (i.e., Saskatchewan Health Authority) who are involved in vaccine rollout and decision-making 5. Large city mayors: Mayors in two cities with the highest population in each province. 6. Public health/public official accounts: Public health professionals working in Ministries of Health, public health agencies, and public health units who are not in categories b) or c); elected public officials (including politicians) who serve in the Cabinet or provincial and local government. 7. Media: Includes media agencies and organizations, and journalists whose tweets are twitter verified for the purpose of broadcasting news. 8. The public: accounts that are not organizational, or without a designated/public official title. |
| Tweet | The body of the tweet, including URL, images, and emoticons. |
| Date posted | Date the tweet was posted. |
| Twitter ID | A unique value assigned to a Twitter account, no two people have the same ID (e.g., @fordnation) |
| Tweet relationship | Quantify who retweeted who using relationships specified in the extraction category (e.g., (a) RT of (b)) |
| Number of replies | The total number of replies since data extraction (e.g., number of responses under the extracted tweet). Having more replies than (retweets + likes) indicate that a tweet may not be viewed as favorably as those with equal numbers of replies and (retweets + likes). |
| Number of retweets | The total number of unquoted retweets since data extraction (e.g., number of retweets as shown in the Twitter text box in image 1). Retweets are broadcasts of a tweet and a form of passive engagement with the person who tweeted, the person who retweets will be noticed by the original tweeter. |
| Number of quote tweets | The total number of retweets with “quotes” (e.g., number of quoted tweets as shown in the Twitter text box, formerly called “retweets with comments” in image 1). Quote tweets broadcast a tweet with added thoughts, and their sentiment tends to correlate with the original tweet sentiment. |
| Total retweet | The combination of “number of retweets” and “number of quote tweets”. This is how we calculate the reply: retweet ratio. |
| Number of likes | The total number of likes since data extraction (e.g., number of likes as shown in the Twitter text box in image 1).  This is used to measure endorsement and used to calculate the reply: like ratio. |
| Number of impressions | The potential number of viewers calculated by summing author and retweeter followers. (Note this is BrandWatch’s metric and different from the “number of impressions” published by Twitter). |
| Does the date of the tweet coincide with any of the policy junctures identified up to three days after the policy juncture? If so, which one? | 1. Yes 2. No |
| Did this tweet reflect or communicate Provincial/Federal policy level changes? | 1. Yes, coincide with NACI statements releases 2. Yes, other policy changes related to vaccine delivery: including any policy changes related to phase changes (e.g., eligibility for vaccination re: age group, gender, special considerations by type of employment or setting) 3. No. |
| Sentiment towards vaccine rollout in the tweet | 1. Positive: Express agreement and endorsement in tweet. Includes positive emoticons, exclamation points, positive adjectives in a tweet, images of people smiling. 2. Negative: express disagreement or doubt in tweet. Includes: negative emoticons, negative adjectives expressing disagreement, anger, frustration, disappointment. 3. Neutral: neither expresses support nor disagreement in tweet. |
