# Supplementary material for: Public Officials’ Engagement on Social Media During the Rollout of the COVID-19 Vaccine: Content Analysis of Tweets
Source: JMIR Infodemiology. 2023 Jul 20;3:e41582. doi: 10.2196/41582 (PMC10361259; doi:10.2196/41582)
Supplement: Multimedia Appendix 6 [file infodemiology_v3i1e41582_app6.docx]

Multimedia Appendix 6. Flow diagram of tweet selection

User accounts

Tweets

Tweets derived mention, reply or retweeted **142** public officials

Tweets derived from 153,200 unique Canadian (geotagged) accounts

Query 2

Query 1

Total number of tweets during full extraction period (December 28, 2020 – August 31, 2021): **602,050**

Total number of tweets driven by public officials across Canada during extraction period: **133,155**

Total number of tweets in top three provinces: **106,834** (Ontario (n=80,201, 75.1%), Alberta (n=15,096, 14.1%) and BC (n=11,537, 10.8%))

Total number of tweets with highest impressions included in content analysis: **270** (30 tweets per three phase changes in each of Ontario, Alberta and BC).
