# Supplementary material for: Public Officials’ Engagement on Social Media During the Rollout of the COVID-19 Vaccine: Content Analysis of Tweets
Source: JMIR Infodemiology. 2023 Jul 20;3:e41582. doi: 10.2196/41582 (PMC10361259; doi:10.2196/41582)

# Multimedia Appendix 7. COVID-19 vaccine administration and case positivity in Alberta, British Columbia, and Ontario (December 2020 to August 2021)

Figure 1. Mention volume of tweets from or to public officials, vaccines administered, and new cases per 100,000 people in Alberta (7-day rolling average). Phase 1, phase 2, and phase 3 (gray lines) indicate the starting date of the vaccine rollout phase change.


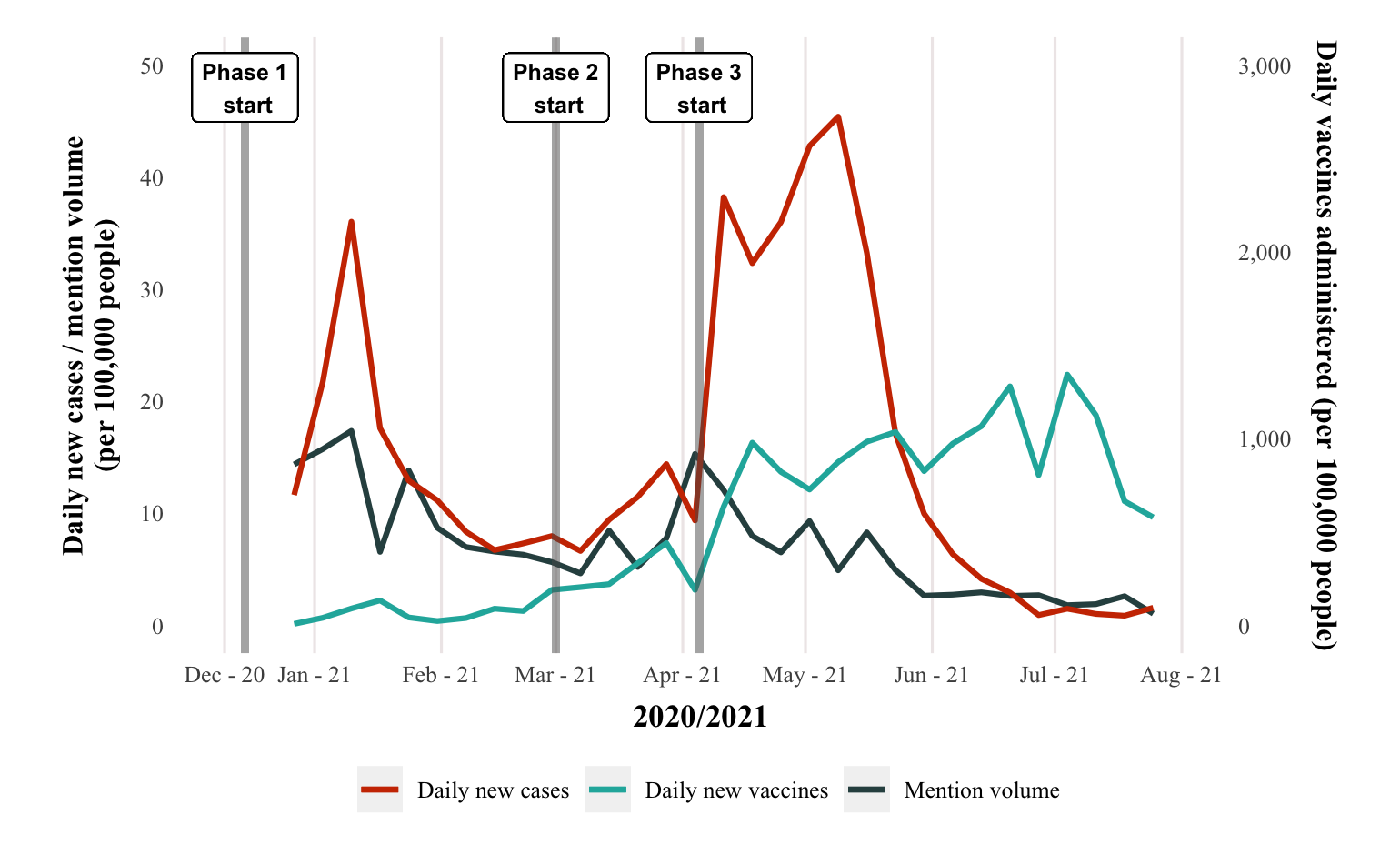


Figure 2. Mention volume of tweets from or to public officials, vaccines administered, and new cases per 100,000 people in British Columbia (7-day rolling average). Phase 1, phase 2, and phase 3 (gray lines) indicate the starting date of the vaccine rollout phase change.


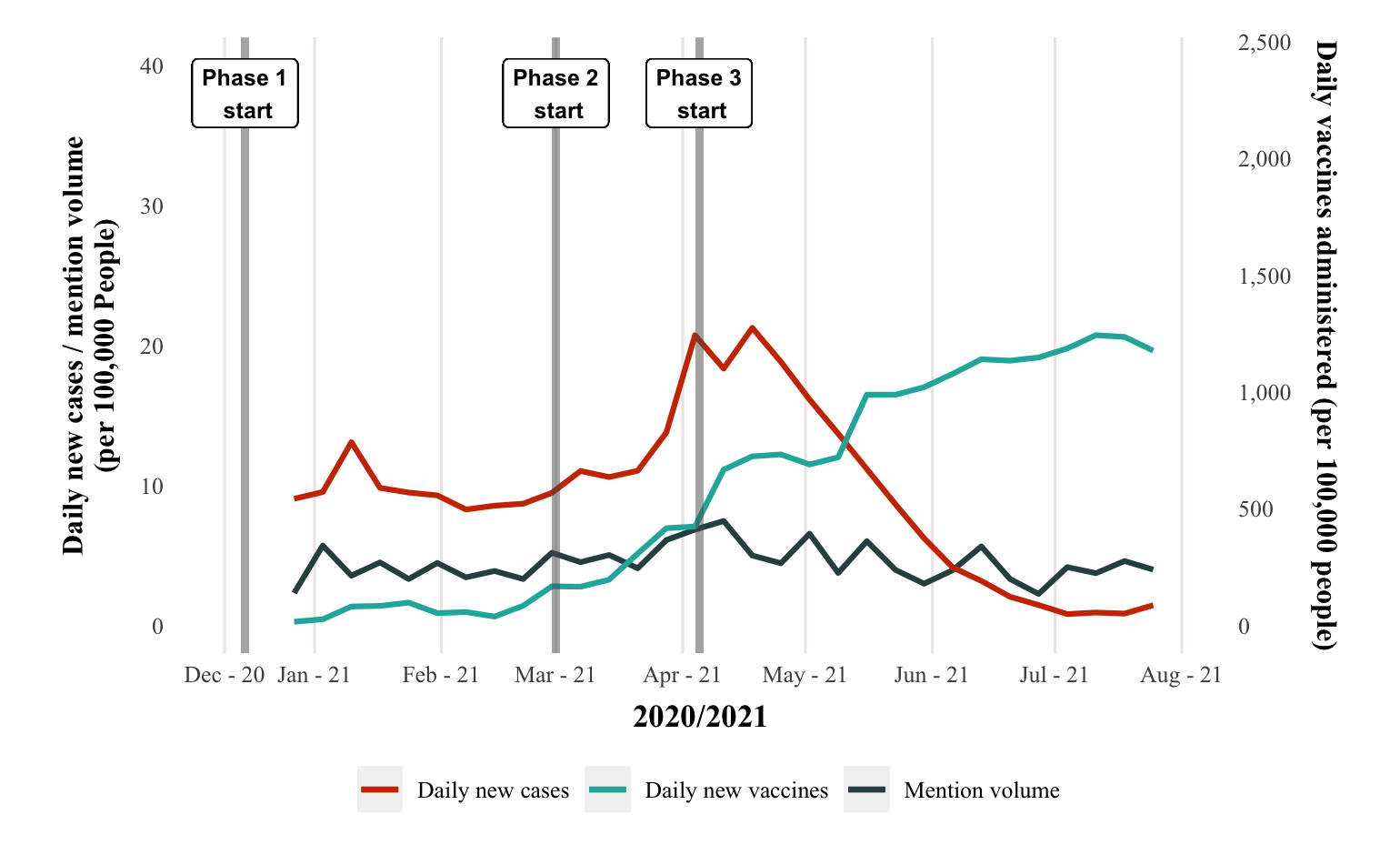


Figure 3. Mention volume of tweets from or to public officials, vaccines administered, and new cases per 100,000 people in Ontario (7-day rolling average). Phase 1, phase 2, and phase 3 (gray lines) indicate the starting date of the vaccine rollout phase change.


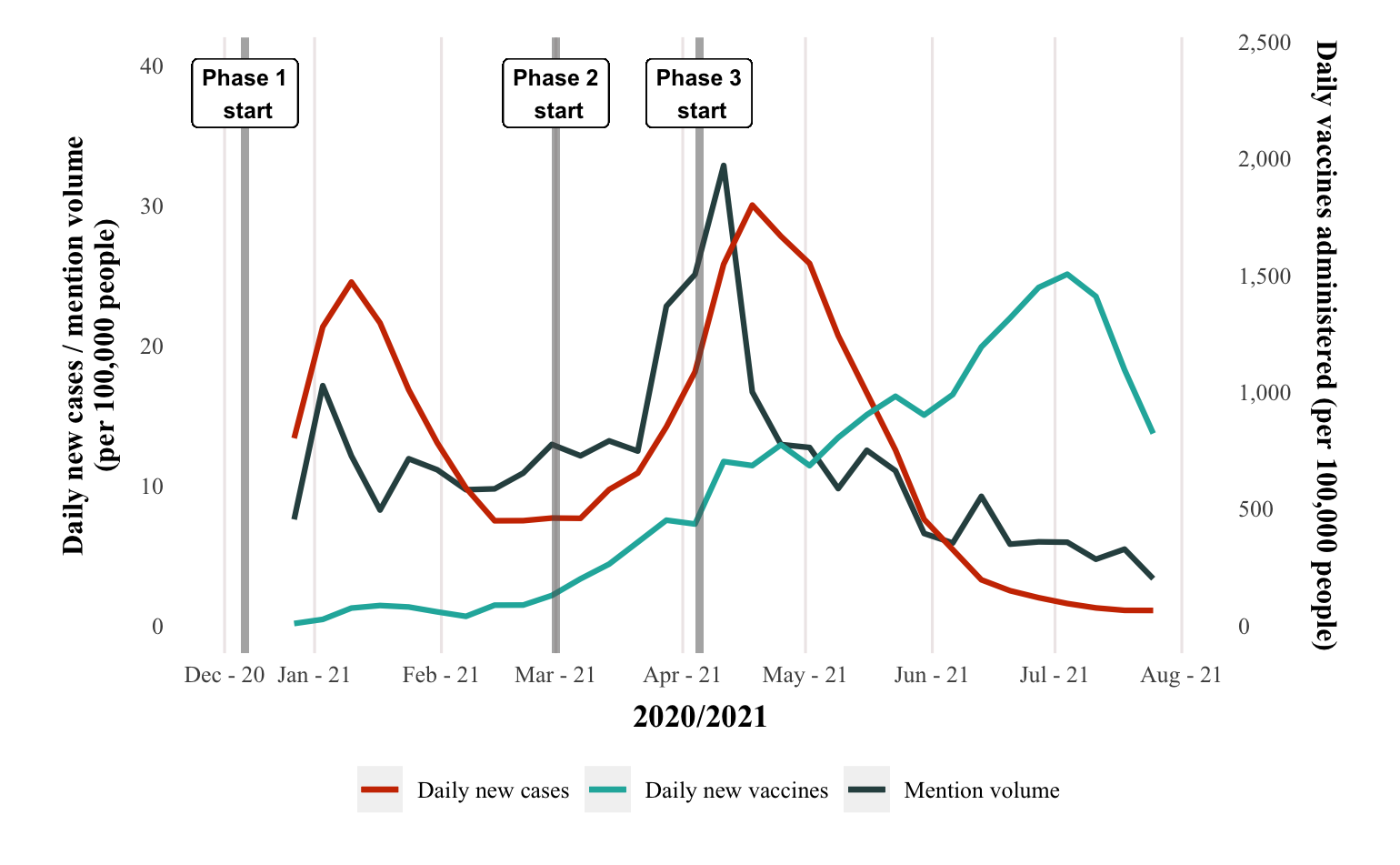

Supplement: Multimedia Appendix 7 [file infodemiology_v3i1e41582_app7.docx]
