# Supplementary material for: Public Officials’ Engagement on Social Media During the Rollout of the COVID-19 Vaccine: Content Analysis of Tweets
Source: JMIR Infodemiology. 2023 Jul 20;3:e41582. doi: 10.2196/41582 (PMC10361259; doi:10.2196/41582)
Supplement: Multimedia Appendix 8 [file infodemiology_v3i1e41582_app8.docx]

# Multimedia Appendix 8. Public officials Twitter engagement metrics in Alberta, British Columbia, and Ontario

| Alberta |  |  |  |  |  |  |  |  |
| --- | --- | --- | --- | --- | --- | --- | --- | --- |
|  | **First Ministers (Premiers)** | **Ministers of Health** | **Chief Medical Officers of Health** | **Government Bodies** | **Largest City Mayors (Municipal Officials)** | **Other Key Public Officials** | **Media** | **Public** |
| Number of tweets | 11 | 9 | 18 | 22 | 1 | 14 | 6 | 9 |
| Avg. Impressions | 282632 | 213041 | 260305 | 186705 | 814695 | 260986 | 191208 | 128776 |
| Avg. retweets | 39 | 16 | 51 | 23 | 0 | 95 | 56 | 119 |
| Avg. Likes | 197 | 43 | 167 | 128 | 82 | 307 | 14 | 317 |
| Avg. Replies | 87 | 28 | 70 | 40 | 0 | 23 | 57 | 48 |
| Like to Impression Ratio | **0.0007** | 0.0002 | 0.0006 | **0.0007** | 0.0001 | **0.0012** | 0.0000 | **0.0025** |
| Push 1 | 6 | 1 | 14 | 9 | 1 | 2 | 6 | 2 |
| Push 2 | 0 | 8 | 0 | 4 | 0 | 0 | 0 | 0 |
| Pull 1 | 1 | 0 | 3 | 3 | 0 | 0 | 0 | 3 |
| Pull 2 | 4 | 0 | 1 | 6 | 0 | 12 | 0 | 4 |
|  |  |  |  |  |  |  |  |  |
| British Columbia |  |  |  |  |  |  |  |  |
|  | **First Ministers (Premiers)** | **Ministers of Health** | **Chief Medical Officers of Health** | **Government Bodies** | **Largest City Mayors (Municipal Officials)** | **Other Key Public Officials** | **Media** | **Public** |
| Number of tweets | 1 | 23 | 0 | 30 | 0 | 18 | 8 | 9 |
| Avg. Impressions | 146795 | 122770 | 0 | 206410 | 0 | 225495 | 104894 | 11055 |
| Avg. retweets | 18 | 18 | 0 | 10 | 0 | 16.8 | 3 | 20 |
| Avg. Likes | 28 | 103 | 0 | 26 | 0 | 2055 | 5 | 131 |
| Avg. Replies | 18 | 18 | 0 | 14 | 0 | 53 | 0 | 14 |
| Like to Impression Ratio | 0.0002 | **0.0008** | N/A | 0.0001 | N/A | **0.0091** | 0.0000 | **0.0118** |
| Push 1 | 1 | 23 | 0 | 28 | 0 | 11 | 8 | 7 |
| Push 2 | 0 | 0 | 0 | 0 | 0 | 0 | 0 | 0 |
| Pull 1 | 0 | 0 | 0 | 2 | 0 | 4 | 0 | 0 |
| Pull 2 | 0 | 0 | 0 | 0 | 0 | 3 | 0 | 2 |
|  |  |  |  |  |  |  |  |  |
| Ontario |  |  |  |  |  |  |  |  |
|  | **First Ministers (Premiers)** | **Ministers of Health** | **Chief Medical Officers of Health** | **Government Bodies** | **Largest City Mayors (Municipal Officials)** | **Other Key Public Officials** | **Media** | **Public** |
| Number of tweets | 12 | 3 | 1 | 19 | 23 | 7 | 19 | 7 |
| Avg. Impressions | 651304 | 4286250 | 657001 | 974306 | 1295619 | 832151 | 1209407 | 729010 |
| Avg. retweets | 133 | 191 | 43 | 57 | 43 | 262 | 249 | 465 |
| Avg. Likes | 594 | 1125 | 103 | 217 | 258 | 957 | 497 | 2362 |
| Avg. Replies | 453 | 133 | 27 | 19 | 31 | 126 | 167 | 107 |
| Like to Impression Ratio | **0.0009** | 0.0003 | 0.0002 | 0.0002 | 0.0002 | **0.0012** | 0.0004 | **0.0032** |
| Push 1 | 5 | 3 | 1 | 12 | 16 | 6 | 17 | 0 |
| Push 2 | 0 | 0 | 0 | 0 | 0 | 0 | 0 | 0 |
| Pull 1 | 3 | 0 | 0 | 2 | 5 | 1 | 2 | 3 |
| Pull 2 | 4 | 0 | 0 | 5 | 2 | 0 | 0 | 4 |
