# Supplementary material for: Public Officials’ Engagement on Social Media During the Rollout of the COVID-19 Vaccine: Content Analysis of Tweets
Source: JMIR Infodemiology. 2023 Jul 20;3:e41582. doi: 10.2196/41582 (PMC10361259; doi:10.2196/41582)
Supplement: Multimedia Appendix 9 [file infodemiology_v3i1e41582_app9.docx]

Multimedia Appendix 9. Examples of tweets conveying positive and negative sentiment concerning vaccine roll-out

| Positive Sentiment (e.g., praise, excitement, support) | *Over 300,000 doses administered in Ottawa! We’re making good progress in our vaccination campaign. Thank you to everyone who has signed up to get their first dose. Great to see so many senior residents, who are most at risk of serious illness, get their vaccine.* – Jim Watson (Mayor of Ottawa), April 23, 2021    *Last year in pictures shows the resilience of @NationalDefence & @CanadianForces members. They helped us through the pandemic, from repatriating 🇨🇦s to working in long-term care facilities, distributing vaccines & persisting through the loss of 9 of their brothers & sisters.* – Harjit Sajjan (Federal Minister of National Defence) – December 31, 2020    *Alberta’s vaccine rollout is one of the best in the country, with among the highest percentage of doses administered. We're also more than doubling the number of pharmacies giving vaccinations, with hundreds more, along with community physicians, in the weeks ahead. 4/5* – Tyler Shandro (Former Minister of Health of Alberta, now MLA), March 24, 2021 |
| --- | --- |
| Negative Sentiment (e.g., frustration, criticism, dismay) | *Earlier this week, it was revealed that UCP had vaccinated only 7,000 Alberta health-care workers when it promised to have administered an initial dose to 29,000 by the end of the year.* – Rachel Notley (Alberta MLA and provincial NDP Leader), December 31, 2020    *What a strange, unequal, haphazard roll out of vaccines is happening in Ontario. Some regions are falling behind, while others are ahead & it's ALL because the Ford govt didn't come up with a plan & dumped it on local PHUs! They only had CLOSE TO A YEAR to plan!? #COVID19Ontario* – Abhijeet Manay (member of public, Deputy Leader of Green Party of Ontario), March 18, 2021    *Today I asked @adriandix if he would commit to improving data transparency moving forward. BC has consistently been one of the least transparent provinces throughout the pandemic - leaving BCers to connect the dots themselves on VOCs & the vaccine roll-out. 1/ #bcpoli #COVID19* – Sonia Furstenau (BC MLA and Provincial Green Party Leader), April 13, 2021    *In the span of about two weeks and after thousands of doses of the AstraZeneca vaccine had been administered, the federal Liberal government has spun in circles on safety advice. Watch this debacle (with date stamps) to see how badly the Liberals have botched this issue.* [*https://t.co/hM4i99Thwz*](https://t.co/hM4i99Thwz) – Michelle Rempel (Federal MP), March 29, 2021    *#BREAKING: @fordnation office: “While we appreciate the Prime Minister’s offer, unless it is matched with an increase in supply, we do not need the Red Cross at this time for the administration of vaccines in Ontario. We do not have a capacity issue, we have a supply issue.”* – Laura Stone (journalist), April 16, 2021    *Hi Jake. Provinces are responsible for the rollout of vaccines. Ontario, run by Conservative Premier @fordnation , currently has 1.2 million vaccines sitting in freezers and only administered 96K yesterday. So that’s a major reason why “so few are vaccinated”. #cdnpoli #onpoli* – Tyler Watt (running for provincial office), April 13, 2021 |
| Neutral (e.g., no stance detected towards vaccine roll-out) | *Premier John Horgan, Minister Adrian Dix, Dr. Bonnie Henry, and Dr. Penny Ballem provide an update on BC's COVID-19 Immunization Plan rollout.* [*#CovidBC*](https://twitter.com/hashtag/CovidBC?src=hashtag_click) – Vancouver Board of Park and Recreation, March 17, 2021  *Ontario is reporting 699 cases of* [*#COVID19*](https://twitter.com/hashtag/COVID19?src=hashtag_click) *and nearly 20,300 tests completed. Locally, there are 207 new cases in Toronto, 144 in Peel, 52 in York Region and 50 in Durham. As of 8:00 p.m. yesterday, 9,202,220 doses of the COVID-19 vaccine have been administered.* –Christine Elliot (Minister of Health in Ontario), June 1, 2021 |
